# Comparative efficacy of trauma scoring for predicting in-hospital mortality in elderly patients in China and Thailand: A multicenter retrospective study

**DOI:** 10.1371/journal.pone.0348074

**Published:** 2026-04-30

**Authors:** Rui Lu, Ploytip Jansiriyotin, Tanyamon Kittidumkerng, Natthida Owattanapanich, Kaweesak Chittawatanarat

**Affiliations:** 1 Department of Surgery, Faculty of Medicine, Chiang Mai University, Chiang Mai, Thailand; 2 Department of Emergency Medicine, The Affiliated Hospital, Southwest Medical University, Luzhou, Sichuan, China; 3 Department of Surgery, Khon Kaen Hospital, Khon Kaen, Thailand; 4 Clinical Surgical Research Center, Department of Surgery, Faculty of Medicine, Chiang Mai University, Chiang Mai, Thailand; 5 Division of Trauma Surgery, Department of Surgery, Faculty of Medicine Siriraj Hospital, Mahidol University, Bangkok, Thailand; Universiti Sains Malaysia, MALAYSIA

## Abstract

**Background:**

With global population aging, trauma prediction models are essential for elderly patients, yet established scoring systems lack validation in Asian populations. This study evaluates clinical characteristics, mortality risk factors, and the predictive efficacy of trauma scoring systems in elderly trauma patients in China and Thailand.

**Methods:**

This multicenter retrospective cohort study included trauma patients aged ≥65 years admitted to four Level 1 trauma centers between 01/01/2023 and 31/12/2023. The analyzed variables included demographics, clinical data, and trauma scores (ISS, NISS, RTS, TRISS, GTOS). Multivariable logistic regression and ROC curve analysis were performed.

**Results:**

Among 963 patients (median age 73 years; 50.6% female), in-hospital mortality was 7.9%. Independent risk factors included age, cancer history, INR, blood transfusion, GCS, and all trauma scores. TRISS had the highest predictive value (AUC = 0.871), followed by GTOS (0.852) and RTS (0.839), all outperforming ISS and NISS.

**Conclusion:**

Age, comorbidities, and trauma scores are significant predictors of in-hospital mortality in elderly trauma patients. TRISS, GTOS, and RTS offer superior prognostic performance, aiding early identification and management of high-risk individuals.

## Background

Given the rapid aging of the global population, the management of elderly trauma patients has become a major challenge for trauma centers. Although clinical management strategies have continuously improved, early identification and evaluation of patient status remain crucial [1,2]. Trauma prediction models not only optimize prehospital transport and the timing of interventions but also contribute to reducing mortality [3,4]. In regions with uneven resource distribution, these models can serve as effective triage tools for the rational allocation of healthcare resources. Additionally, providing palliative care for this vulnerable population may further enhance the quality of life [5].

Currently, trauma scoring systems can be broadly categorized into three types: anatomical, physiological, and combined scores, each with distinct applicability in prehospital and in-hospital settings. However, given the multifactorial nature of clinical decision-making [6,7], there is a need for accurate and timely prognostic tools.

Although scoring systems such as the Injury Severity Score (ISS) [8], New Injury Severity Score (NISS) [9], Revised Trauma Score (RTS) [10], Trauma and Injury Severity Score (TRISS), and Geriatric Trauma Outcome Score (GTOS) have been extensively validated in elderly populations in Western cohorts [11–13], their performance in Asian populations—particularly in China and Thailand—remains underexplored. Variations in healthcare systems and injury patterns across regions may lead to differences in model performance. Moreover, conventional indices may not fully capture the biological complexity of elderly patients, underscoring the importance of incorporating additional clinical variables and regional validation. Therefore, this study aims to identify risk factors for in-hospital mortality among elderly trauma patients and to assess the predictive performance of various trauma scoring systems in this population.

## Methods

### Study design and data sources

This retrospective study enrolled elderly trauma patients admitted between 01/01/2023 and 31/12/2023 to four trauma centers: the Affiliated Hospital of Southwest Medical University in China (serving approximately 60 million people in Sichuan and surrounding areas), and three hospitals in Thailand—Maharaj Nakorn Chiang Mai Hospital (covering 16 provinces in northern Thailand), Khon Kaen Hospital (serving upper part of northeastern Thailand), and Siriraj Hospital (serving Bangkok Metropolitan Region). Data were accessed for research purposes on 20/06/2024 for the Affiliated Hospital of Southwest Medical University and Maharaj Nakorn Chiang Mai Hospital, and on 15/03/2025 for Khon Kaen Hospital and Siriraj Hospital. The study strictly followed the STROBE statement [14] and received approval from the ethics committees of the participating institutions: the Clinical Trial Ethics Committee of the Affiliated Hospital of Southwest Medical University; the Research Ethics Committee of the Faculty of Medicine, Chiang Mai University (for Maharaj Nakorn Chiang Mai Hospital); the Khon Kaen Hospital Institute Review Board in Human Research; and the Siriraj Institutional Review Board (for Siriraj Hospital). Given the retrospective observational design of the study, informed consent was waived by all four ethics committees.

### Inclusion and exclusion criteria

Inclusion criteria: Patients aged >65 years admitted under trauma care service, with no restriction on ISS. patients were included regardless of whether they received surgical or non-surgical management during hospitalization. Exclusion criteria: (1) hospital-acquired trauma; (2) incomplete data; (3) no vital signs upon admission or death in the emergency department; (4) discharge against medical advice or unknown outcomes. Patients with missing key variables required for the calculation of trauma scores or primary analyses were excluded; the proportion of missing data was low (<5%), and therefore no imputation was performed.

### Study variables

Data collection encompassed demographics, trauma type and mechanism, preexisting comorbidities (S1 Table), initial vital signs (heart rate, respiratory rate, blood pressure, shock index, Glasgow Coma Scale [GCS]), laboratory parameters (hemoglobin, lactate, white blood cell count, thrombocytes, hematocrit, international normalized ratio (INR), creatinine, bilirubin, sodium and potassium), trauma scores (ISS, NISS, RTS, TRISS, GTOS), as well as the modified frailty index (mFI-5) [15]. Hospital complications were recorded and classified into the following categories: pneumonia, urinary tract infection (UTI), sepsis, wound infection and others.

### Statistical analysis

Statistical analyses were conducted using Stata 17.0. Continuous variables were tested for normality using the Shapiro-Wilk and skewness-kurtosis tests, and expressed as mean ± standard deviation (SD) or median (IQR) as appropriate; categorical variables were summarized as frequencies (percentages). Group comparisons employed Student’s t-test or the Mann-Whitney U test for continuous variables and Pearson’s chi-square or Fisher’s exact test for categorical variables. Variables significant on univariate analysis were entered into a multivariable logistic regression model, with results reported as odds ratios (OR) and 95% confidence intervals (CI). The predictive performance of the scoring systems was assessed via the area under the receiver operating characteristic (ROC) curve (AUC) [16], with predicted probabilities transformed back to the original scoring variables [17]. Differences between models were evaluated using DeLong’s test [18], and a *p*-value of less than 0.05 was defined as statistically significant.

## Results

A total of 963 elderly trauma patients were enrolled. Among these, 624 (64.8%) patients were from the Affiliated Hospital of Southwest Medical University (China), 205 (21.3%) from Khon Kaen Hospital (Thailand), 84 (8.7%) from Maharaj Nakorn Chiang Mai Hospital (Thailand), and 50 (5.2%) from Siriraj Hospital (Thailand). The median age was 73 [(IQR) 68–80] years, with an almost equal sex distribution (50.6% female, 49.4% male), and predominantly blunt injuries (97.6%). The median ISS was 9 [5–14], as shown in Table 1. Low-energy falls (≤2 meters) were the predominant injury mechanisms (62%), followed by pedestrian traffic accidents (11.5%). The anatomic distribution of severe injuries (AIS ≥ 3) among geriatric trauma patients demonstrated that lower extremity trauma was the most prevalent type of injury, accounting for 24.1% of cases, followed by head injuries, which accounted for 20.5% of cases (S2 Table).

**Table 1:** Demographics and hospital data.

Variables	Total (N = 963)	Survival group (N = 887)	Non-survival group (N = 76)	*P*
**Demographics**				
Age, median (IQR), y	73 (68–80)	73 (68–80)	74.5 (70–79.5)	0.487
65–74 y, n (%)	528 (54.8)	490 (55.2)	38 (50.0)	0.165
75–84 y, n (%)	324 (33.6)	292 (32.9)	32 (42.1)	
≥ 85 y, n (%)	111 (11.5)	105 (11.8)	6 (7.9)	
Male	476 (49.4)	429 (48.4)	47 (61.8)	0.024
**Mechanism of injury, n (%)**				0.009
Low level fall ≤ 2m	597 (62.0)	564 (63.6)	33 (43.4)	
High level fall > 2m	48 (5.0)	44 (5.0)	4 (5.3)	
Motor-cycle	74 (7.7)	61 (6.9)	13 (17.1)	
Motor vehicle	64 (6.7)	56 (6.3)	8 (10.5)	
Pedestrian	111 (11.5)	97 (10.9)	14 (18.4)	
Blast injury	2 (0.2)	2 (0.2)	0 (0)	
Stab wound	5 (0.5)	5 (0.6)	0 (0)	
Assaulted	2 (0.2)	2 (0.2)	0 (0)	
Other	60 (6.2)	56 (6.3)	4 (5.3)	
**Vital signs on arrival (1st)**				
SBP < 90 mm-Hg,n (%)	25 (2.6)	17 (1.9)	8 (10.5)	< 0.001
Heart rate: bpm, median (IQR)	81 (74–92)	80 (73–90)	90.5 (79.5–102)	< 0.001
Respiratory rate: bpm, median (IQR)	20 (20–20)	20 (20–20)	19 (0–20)	< 0.001
SI, median (IQR)	0.59(0.50–0.68)	0.58(0.5–0.67)	0.65 (0.53–0.80)	0.002
SI ≥ 1, n (%)	36 (3.7)	26 (2.9)	10 (13.2)	< 0.001
**Scoring systems**				
GCS, median (IQR)	15 (15–15)	15 (15–15)	6.5 (3–14)	< 0.001
ISS, median (IQR)	9 (5–14)	9 (5–10)	25 (11.5–33)	< 0.001
ISS (1–9), n (%)	661 (68.6)	644 (72.6)	17 (22.4)	< 0.001
ISS (10–15), n (%)	84 (8.7)	80 (9.0)	4 (5.3)	
ISS (16–24), n (%)	113 (11.7)	99 (11.2)	14 (18.4)	
ISS (≥25), n (%)	105 (10.9)	64 (7.2)	41 (54.0)	
NISS, median (IQR)	9 (5–17)	9 (5–14)	25.5 (15–38)	< 0.001
RTS, median (IQR)	7.84 (7.84–7.84)	7.84 (7.84–7.84)	5.39 (4.09–7.70)	< 0.001
TRISS (%), median (IQR)	96.75 (95.15–97.97)	96.75 (96.48–98.29)	75.67 (30.79–92.63)	< 0.001
GTOS, median (IQR)	98.5 (88.5–113.5)	97 (87.5–110)	139 (114.5–167.5)	< 0.001
mFI-5, median (IQR)	1 (0–2)	1 (0–2)	1 (0–2)	0.463
**Laboratory 1**^**st**^ **test results**				
Hemoglobin(g/L), median (IQR)	114 (97–126)	114 (100–126)	100.5 (82.5–118)	< 0.001
Leucocytes(10^9/L), median (IQR)	8.83 (6.9–12.04)	8.6 (6.77–11.35)	13.25 (9.08–17.96)	< 0.001
Thrombocytes(10^9/L), median (IQR)	193 (148–247)	197 (151–248)	160 (103–206.5)	< 0.001
Hematocrit, median (IQR)	0.35 (0.3–0.38)	0.35 (0.31–0.39)	0.31 (0.25–0.37)	< 0.001
INR, median (IQR)	1.03 (0.97–1.1)	1.02 (0.96–1.09)	1.13 (1.04–1.51)	< 0.001
Creatinine(μmol/L), median (IQR)	73.7 (59.23–94.7)	73.1 (58.8–93.8)	81.78 (67.35–111.6)	0.004
Bilirubin (TBIL, μmol/L), median (IQR)	12.48 (8.89–17.6)	12.48 (8.9–17.44)	12.85 (7.0–19.45)	0.545
Na (mmol/L), median (IQR)	139.6 (137–141.7)	139.6 (137.1–141.7)	138.65 (136–141.9)	0.228
K (mmol/L), median (IQR)	3.84 (3.56–4.2)	3.85 (3.58–4.2)	3.7 (3.39–4.21)	0.183
**Any Comorbidities, n (%)**	738 (76.6)	686 (77.3)	52 (68.4)	0.078
**Clinical outcomes**				
Blood transfusion within 24h, n (%)	103 (10.7)	67 (7.6)	36 (47.4)	< 0.001
ICU admission, n (%)	185 (19.2)	127 (14.3)	58 (76.3)	< 0.001
Any In-hospital complication, n (%)	284 (29.5)	250 (28.2)	34 (44.7)	0.002
Hospitalization days (LOS), median (IQR)	8 (3–14)	8 (3–15)	3 (1–9.5)	< 0.001
ICU days (n = 185), median (IQR)	4 (1–9)	4 (1–11)	2 (1–7)	0.096

Abbreviations: SBP: Systolic blood pressure; SI: shock index; GCS, Glasgow Coma Score; ISS: Injury Severity Score; NISS: New Injury Severity Score; RTS: Revised Trauma Score; TRISS: Trauma and Injury Severity Score; GTOS: Geriatric Trauma Outcome Score; mFI-5: modified frailty index-5; LOS: Length of Stay; ICU: Intensive Care Unit.

Regarding comorbidities, 76.6% of patients had at least one underlying condition, predominantly hypertension (47.5%), osteoporosis (29.2%), and diabetes mellitus (20.2%). During hospitalization, 29.9% of patients developed complications (primarily pneumonia), and 19.2% required ICU admission (median ICU stay: 4 [1–9] days).

Intergroup analysis revealed that the non-survival group had a higher proportion of males and a significantly greater incidence of traffic accident-related deaths. In contrast, the survival group demonstrated more favorable physiological parameters, including higher GCS, hemoglobin, thrombocytes, and hematocrit, and lower heart rate, white blood cell count, SI, INR, and creatinine (all *p* < 0.05). Additionally, the non-survival group experienced a markedly elevated transfusion rate (*p* < 0.001) and a higher complication rate. No significant differences were found in serum sodium, potassium levels, or the overall prevalence of comorbidities.

The median hospital length of stay (LOS) was 8 [3–14] days, with an overall mortality rate of 7.9% and a median time interval between admission and death was 3 [1–9.5] days. Although the intensive care unit (ICU) stay duration did not differ significantly between groups, the non-survival group had a shorter total hospital stay (*p* < 0.001).

Multivariable logistic regression analysis (Table 2) identified increased age (aOR = 1.06), prior cancer history (aOR = 6.98), elevated INR (aOR = 2.96), transfusion (aOR = 2.26), higher GCS (aOR = 0.70) and all trauma scores (ISS, NISS, RTS, TRISS, GTOS) as independent risk factors for in-hospital mortality..

**Table 2:** Multivariate analysis of factors for mortality.

Variable	aOR (95%CI)	*p*
Age	1.06 (1.01–1.11)	0.015
Cancer history	6.98 (1.94–25.15)	0.007
INR	2.96 (1.51–5.82)	0.002
Blood transfusion within 24 hours	2.26 (1.03–4.96)	0.043
GCS	0.70 (0.63–0.77)	< 0.001
ISS	1.11 (1.07–1.15)	< 0.001
NISS	1.06 (1.03–1.09)	< 0.001
RTS	0.31 (0.23–0.41)	< 0.001
TRISS	0.01 (0.00–0.02)	< 0.001
GTOS	1.04 (1.03–1.06)	< 0.001

Abbreviations: aOR: Adjusted Odds Ratio; CI: Confidence Interval; INR, International normalized ratio; GCS, Glasgow Coma Score;.ISS: Injury Severity Score; NISS: New Injury Severity Score; RTS: Revised Trauma Score; TRISS: Trauma and Injury Severity Score; GTOS: Geriatric Trauma Outcome Score.

**Fig 1:**
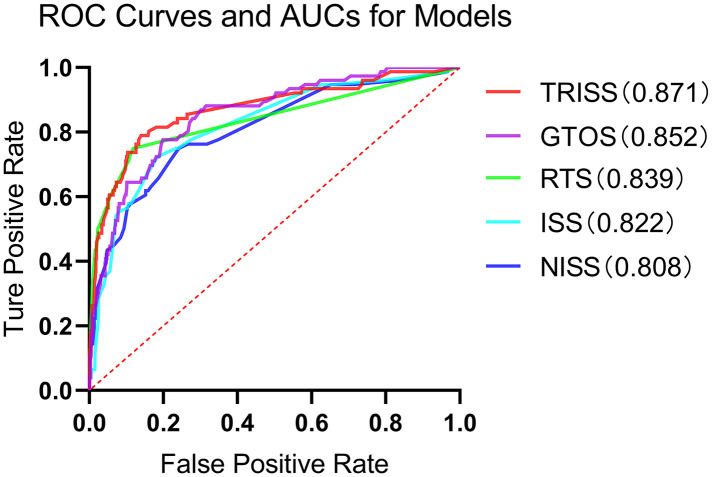
ROC curves of ISS, NISS, RTS, TRISS, and GTOS for predicting mortality in elderly trauma patients.

To assess the comparative predictive efficacy of various trauma scores for mortality, ROC curves (Fig 1) were generated based on survival probabilities derived from ISS, NISS, RTS, TRISS, and GTOS.

The AUC and 95% CIs for each scoring system are presented in Table 3. Among them, TRISS demonstrated the highest discriminative ability (AUC = 0.871), followed by GTOS (AUC = 0.852) and RTS (AUC = 0.839).

**Table 3:** Area under the ROC curve and confidence intervals for the predictive performance of each trauma scoring system.

Trauma score	AUC (95%CI)
ISS	0.822 (0.770–0.874)
NISS	0.808 (0.752–0.863)
RTS	0.839 (0.786–0.892)
TRISS	0.871 (0.821–0.921)
GTOS	0.852 (0.807–0.898)

Abbreviations: ROC: receiver operating characteristic; AUC: area under the receiver operating characteristic curve; CI: confidence interval; ISS: Injury Severity Score; NISS: New Injury Severity Score; RTS: Revised Trauma Score; TRISS: Trauma and Injury Severity Score; GTOS: Geriatric Trauma Outcome Score.

Table 4 presents the optimal cut-off values for each trauma scoring system, along with the corresponding sensitivity, specificity, accuracy, and predictive values. TRISS (cut-off = 0.93) exhibited the highest sensitivity (78.9%) and specificity (86.0%), whereas RTS (cut-off = 7.68) showed the highest specificity (88.2%) with comparable sensitivity (75.0%).

**Table 4:** Optimal cut-off values for each trauma score with corresponding sensitivity, specificity, accuracy, and predictive values.

Trauma score	Cut-off value	Sensitivity (%)	Specificity (%)	Accuracy (%)	PPV (%)	NPV (%)
ISS	15	72.4	81.6	77	25.2	97.2
NISS	15	75	75.9	75	21	97.3
RTS	7.68	75	88.2	82	35.2	97.6
TRISS	0.93	78.9	86	82	32.6	97.9
GTOS	113.75	77.6	80.2	79	25.1	97.7

Abbreviations: ISS: Injury Severity Score; NISS: New Injury Severity Score; RTS: Revised Trauma Score; TRISS: Trauma and Injury Severity Score; GTOS: Geriatric Trauma Outcome Score.

PPV: Positive predictive value, NPV: Negative predictive value.

Multivariable logistic regression analysis (Table 5) confirmed that ISS ≥ 15 (aOR = 4.23), NISS ≥ 15 (aOR = 2.83), RTS ≤ 7.68 (aOR = 9.77), TRISS ≤ 0.93 (aOR = 8.01), and GTOS ≥ 113.75 (aOR = 6.10) were independent predictors of mortality.

**Table 5:** Multivariable analysis of optimal cutoff values of trauma scores for mortality risk.

Trauma Score	Cut-off Value	aOR (95% CI)	*p*
ISS	≥ 15	4.23 (1.92–9.32)	< 0.001
NISS	≥ 15	2.83 (1.27–6.31)	0.011
RTS	≤ 7.68	9.77 (4.57–20.89)	< 0.001
TRISS	≤ 0.93	8.01 (3.67–17.47)	< 0.001
GTOS	≥ 113.75	6.10 (2.85–13.05)	< 0.001

Abbreviations: aOR: Adjusted Odds Ratio; CI: Confidence Interval; ISS: Injury Severity Score; NISS: New Injury Severity Score; RTS: Revised Trauma Score; TRISS: Trauma and Injury Severity Score; GTOS: Geriatric Trauma Outcome Score.

**Fig 2:**
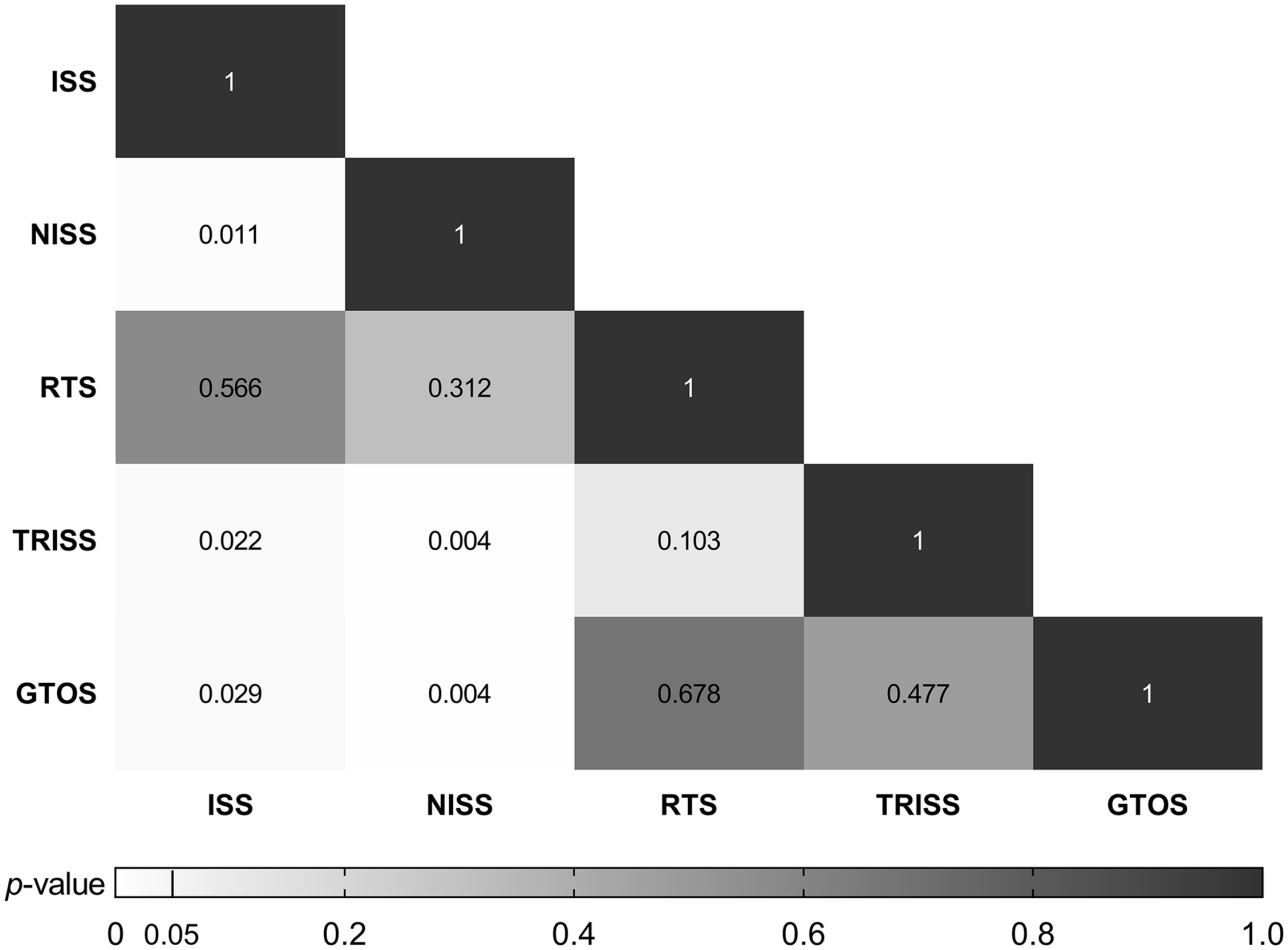
Comparison of AUCs of different scoring systems (using *p*-values).

Comparison of AUCs (Fig 2) revealed that TRISS significantly outperformed ISS and NISS (*p* < 0.05) but showed no significant differences compared to GTOS (*p* = 0.477) or RTS (*p* = 0.103). GTOS also demonstrated superior predictive performance over ISS and NISS (*p* < 0.05) but was not significantly different from RTS (*p* = 0.678). Although RTS had a relatively high AUC, it did not significantly differ from ISS or NISS (*p* > 0.05). Overall, TRISS, GTOS, and RTS demonstrated comparable predictive performance, with no statistically significant differences observed among them, despite TRISS having the highest AUC. These findings suggest similar rather than clearly superior discriminative ability among these scoring systems.

## Discussion

In this multicenter study conducted in China and Thailand, we examined the associations between initial clinical indicators and in-hospital mortality among elderly trauma patients and compared the predictive performance of commonly used trauma scoring systems. Although earlier studies have shown that the overall mortality rate for adult trauma patients declined from 8.8% in 1995 to 4.9% in 2008 [19], mortality rates among elderly trauma patients remain as high as 12.85% in England and 9.6% in the United States [20,21]. However, the in-hospital mortality rate of elderly trauma patients in our study was 7.9%. This difference may be explained by the lower median ISS in our study compared to previous studies (9 vs. 14.8).

Consistent with previous research findings [22,23], we found that falls and vehicle accidents were the main mechanisms of injury, with the lower extremities and head being the most frequently affected areas. Multivariable logistic regression identified increasing age, a history of cancer, elevated INR, and blood transfusion within 24 hours as independent predictors of mortality. Mortality positively correlated with increasing age, a finding consistent with previous studies [12]. A history of cancer may reduce physiological reserve [24], while elevated INR, and early transfusion likely reflect severe injury, significant blood loss, or acute traumatic coagulopathy [25,26]. Although heart rate and shock index differed between survivors and non-survivors, they did not retain significance in multivariable models, possibly due to diminished physiological responsiveness in elderly patients [27]. A study by Andrea et al. similarly demonstrated the limited utility of the shock index in predicting mortality among elderly trauma patients [28].

Although recent studies have indicated that frailty significantly impacts mortality in elderly trauma patients [12,29], this study did not observe a significant association between the mFI-5 and in-hospital mortality. This may be attributable to the relatively low frailty burden in our cohort, as evidenced by a median mFI-5 score of 1 [0–2], which may have limited its discriminative ability for mortality prediction.

Research has shown that although the ISS is widely recognized as the gold standard for anatomical injury assessment, it exhibits notable limitations in elderly trauma populations. The ISS does not account for age-related factors or physiological parameters, resulting in an inadequate evaluation of physiological vulnerability and comorbidity burden in older patients [30]. Notably, although the NISS optimizes polytrauma assessment by incorporating the three most severe injuries regardless of location [31], its predictive performance in our study was inferior to that of the ISS. This finding contrasts with that of Jiang et al., who reported AUC values of 0.807, 0.850, and 0.828 for ISS, NISS, and TRISS, respectively, with NISS demonstrating the highest predictive accuracy [32]. This divergence may be attributed to population heterogeneity: in our study, low-energy falls predominated (61.5%), and the incidence of high-energy polytrauma was significantly lower than in general trauma populations [33], thereby limiting the advantage of NISS.

In contrast, the RTS, which relies exclusively on dynamic physiological parameters (including systolic blood pressure, and respiratory rate) and GCS to reflect real-time physiological reserve [34], demonstrated predictive value comparable to that of the TRISS and the GTOS (*p* > 0.05). This suggests that mortality in elderly trauma patients may be more strongly driven by acute physiological decompensation than by anatomical injury alone.

The superior performance of TRISS and GTOS is attributable to their multidimensional evaluation framework: TRISS integrates both physiological and anatomical parameters, while GTOS combines age, ISS, and transfusion requirements [35]. However, TRISS may be sensitive to variations in prehospital care and time to hospital presentation due to its reliance on multiple physiological parameters [36]. In contrast, GTOS may be influenced by clinical decision-making processes and potential delays in care.

A recent meta-analysis further confirmed the superiority of TRISS, with a combined AUROC of 0.82, which is higher than that of ISS (0.74) and GTOS (0.80) [30], which is consistent with our findings. However, Park et al. reported that GTOS outperformed TRISS in predicting mortality among trauma patients aged 65 and older [37], while Barea-Mendoza et al. found that GTOS was inferior to TRISS in this context [38]. These inconsistencies suggest that the performance of predictive models may vary across populations and study settings, and further investigation is needed to ascertain their clinical applicability and value.

Although ISS and NISS performed worse than TRISS, GTOS, and RTS, they still demonstrated strong predictive value, with AUCs above 0.8. We also identified optimal cut-off values for trauma severity scores. Multivariable analysis confirmed these thresholds as independent predictors of mortality, with adjusted odds ratios (aORs) ranging from 2.83 to 9.77. These criteria may facilitate simplified risk stratification compared with traditional scoring approaches. Clinicians should prioritize intensive monitoring and targeted interventions when patients meet or exceed the following thresholds: TRISS (≤ 0.93), GTOS (≥ 113.75), RTS (≤ 7.68), and ISS/NISS (≥ 15). Overall, TRISS, GTOS and RTS demonstrated superior performance in risk stratification among elderly trauma patients.

The strengths of this study include its large multicenter design and the comprehensive evaluation of injury characteristics and early clinical indicators, which provide valuable insights for the timely identification of high-risk patients. However, several limitations should be acknowledged. The retrospective design may introduce inherent bias, and the absence of 30-day post-discharge follow-up limits the assessment of longer-term outcomes, which may also be influenced by non-traumatic factors. Although the proportion of missing data was low, the exclusion of patients with incomplete key variables may have introduced potential selection bias. In addition, variations in data collection timelines across participating centers, due to differences in institutional approval processes, may have resulted in minor heterogeneity in data acquisition and recording practices. The generalizability of our findings may also be limited, as this study was conducted in four tertiary trauma centers and may not fully represent other healthcare settings, particularly rural or resource-limited environments. Furthermore, although frailty is an important determinant of outcomes in elderly trauma patients, no significant association between mFI-5 and mortality was observed in our study. This may be explained by the relatively low frailty burden in our cohort or the limited sensitivity of mFI-5 in capturing frailty-related risk, particularly in patients with fracture-dominant injuries. Similarly, we did not identify significant associations between mortality and some previously reported risk factors (such as male sex, diabetes mellitus, and chronic renal failure), possibly due to limited statistical power or heterogeneity in patient characteristics [39]. Therefore, the findings should be interpreted with caution. Future prospective studies are warranted to further validate TRISS, GTOS, and RTS, and to develop more comprehensive predictive models to optimize management and improve outcomes in elderly trauma patients.

## Conclusion

Multiple factors are associated with mortality in elderly patients, and all trauma scoring systems show a good correlation with mortality in China and Thailand geriatric populations. TRISS, GTOS, and RTS demonstrate superior predictive performance for mortality in elderly trauma patients, facilitating the early detection of high-risk individuals and optimizing clinical management strategies.

## Supporting information

S1 Table Comorbidities and their academic descriptions. (DOCX)

S2 Table Detailed clinical characteristics of elderly trauma patients including severe injury distribution, comorbidities, and in-hospital complications. (DOCX)
