# Supplementary material for: Comparative efficacy of trauma scoring for predicting in-hospital mortality in elderly patients in China and Thailand: A multicenter retrospective study
Source: PLoS One. 2026 Apr 30;21(4):e0348074. doi: 10.1371/journal.pone.0348074 (PMC13132200; doi:10.1371/journal.pone.0348074)
Supplement: S1 Table — (DOCX) [file pone.0348074.s001.docx]

S1 Table. Comorbidities and their academic descriptions

| **Comorbidities** | **Description** |
| --- | --- |
| Coronary artery disease | Documented history of ischemic heart disease (IHD) or previous cardiac interventions specifically addressing IHD. |
| Anticoaguant/antiplatelet use | Patients currently receiving anticoagulation therapy (e.g., low-molecular-weight heparin [LMWH], warfarin, or direct oral anticoagulants) or antiplatelet agents (including aspirin). |
| Diabetic mellitus | Patients requiring insulin or oral antihyperglycemic agents for glycemic control. |
| Hypertension | Patients with a documented history of hypertension necessitating pharmacological treatment. |
| Chronic renal failure | Patients with preexisting renal impairment, including those requiring dialysis. |
| COPD or Asthma | Patients with a documented diagnosis or currently receiving treatment for chronic obstructive pulmonary disease or asthma. |
| Cirrhosis | Patients with documented cirrhosis or advanced liver disease. |
| Neurological diseases | Patients with a history of cerebrovascular accident (CVA), severe parkinsonism, or those on antiepileptic therapy. |
| Dementia | Patients with an established clinical diagnosis of dementia. |
| Cancer history | Patients with a confirmed history of malignancy, either undergoing treatment or in remission. |
| Visual disorders | Clinically significant impairment in the sense of vision. |
| Osteoporosis | Documented osteoporosis or clinically identified risk factors contributing to bone fragility. |
| Others | Any other clinically significant comorbidities not otherwise classified (e.g., HIV). |
