# Supplementary material for: Comparative efficacy of trauma scoring for predicting in-hospital mortality in elderly patients in China and Thailand: A multicenter retrospective study
Source: PLoS One. 2026 Apr 30;21(4):e0348074. doi: 10.1371/journal.pone.0348074 (PMC13132200; doi:10.1371/journal.pone.0348074)
Supplement: S2 Table — (DOCX) [file pone.0348074.s002.docx]

S2 Table. Detailed clinical characteristics of elderly trauma patients including severe injury distribution, comorbidities, and in-hospital complications

| **Variables** | **Total**  **（N=963）** | **Survival group**  **(N=887)** | **Non-survival group**  **(N=76)** | *P* |
| --- | --- | --- | --- | --- |
| **AIS ≥3, n** (%) |  |  |  |  |
| Head AIS ≥3 | 197 (20.5) | 145 (16.3) | 52 (68.4) |  |
| Face AIS ≥3 | 3 (0.3) | 3 (0.3) | 0 (0) |  |
| Neck AIS ≥3 | 23 (2.4) | 21 (2.4) | 2 (2.6) |  |
| Thorax AIS ≥3 | 119 (12.4) | 102 (11.5) | 17 (22.4) |  |
| Abdomen AIS ≥3 | 37 (3.8) | 31 (3.5) | 6 (7.9) |  |
| Spine AIS ≥3 | 111 (11.5) | 100 (11.3) | 11 (14.5) |  |
| Upper Extremity AIS ≥3 | 13 (1.3) | 12 (1.4) | 1 (1.3) |  |
| Lower Extremity AIS ≥3 | 232 (24.1) | 222 (25.0) | 10 (13.2) |  |
| External and other AIS ≥3 | 14 (1.5) | 9 (1.0) | 5 (6.6) |  |
| Multiple AIS≥3 | 118 (12.3) | 94 (10.6) | 24 (31.6) |  |
| **Comorbidities, n (%)** |  |  |  |  |
| Coronary artery disease | 71 (7.4) | 67 (7.6) | 4 (5.3) | 0.647 |
| Anticoagulant/antiplatelet use | 44 (4.6) | 41 (4.6) | 3 (4.0) | 1 |
| Diabetic mellitus | 195 (20.3) | 176 (19.8) | 19 (25.0) | 0.283 |
| Hypertension | 457 (47.5) | 427 (48.1) | 30 (39.5) | 0.146 |
| Chronic renal failure | 39 (4.1) | 33 (3.7) | 6 (7.9) | 0.076 |
| COPD or Asthma | 154 (16.0) | 149 (16.8) | 5 (6.6) | 0.02 |
| Cirrhosis | 3 (0.3) | 2 (0.2) | 1 (1.3) | 0.219 |
| Neurological diseases | 112 (11.6) | 102 (11.5) | 10 (13.2) | 0.665 |
| Dementia | 17 (1.8) | 16 (1.8) | 1 (1.3) | 1 |
| Cancer history | 49 (5.1) | 43 (4.9) | 6 (7.9) | 0.246 |
| Visual disorders | 7 (0.7) | 6 (0.7) | 1 (1.3) | 0.439 |
| Osteoporosis | 281 (29.2) | 275 (31.0) | 6 (7.9) | < 0.001 |
| Others | 82 (8.5) | 72 (8.1) | 10 (13.2) | 0.131 |
| **Number of comorbidities, n (%)** |  |  |  | 0.428 |
| 0 | 225 (23.4) | 201 (22.7) | 24 (31.6) |  |
| 1 | 328 (34.1) | 304 (34.3) | 24 (31.6) |  |
| 2 | 202 (21.0) | 186 (21.0) | 16 (21.1) |  |
| 3 | 104 (10.8) | 98 (11.1) | 6 (7.9) |  |
| 4 | 77 (8.0) | 73 (8.2) | 4 (5.3) |  |
| 5 | 23 (2.4) | 22 (2.5) | 1 (1.3) |  |
| 6 | 4 (0.4) | 3 (0.3) | 1 (1.3) |  |
| **In hospital complication, n (%)** |  |  |  | 0.002 |
| Pneumonia | 155 (16.1) | 142 (16.0) | 13 (17.1) | 0.803 |
| UTI | 35 (3.6) | 32 (3.6) | 3 (4.0) | 0.879 |
| Sepsis | 24 (2.5) | 19 (2.1) | 5 (6.6) | 0.017 |
| Wound infection | 35 (3.6) | 28 (3.2) | 7 (9.2) | 0.007 |
| Other | 154 (16.0) | 129 (14.5) | 25 (32.9) | < 0.001 |

Abbreviations: AIS: Abbreviated Injury Scale; UTI: Urinary tract infection;
